# Complications and Influential Perioperative Factors Associated with SpaceOAR Hydrogel Placement

**DOI:** 10.1155/2024/3439727

**Published:** 2024-09-03

**Authors:** Kerith R. Wang, Rishabh K. Simhal, Cassra B. Clark, Mark J. Mann, James R. Mark, Costas D. Lallas, Robert Den, Edouard J. Trabulsi

**Affiliations:** ^1^ Department of Urology Sidney Kimmel Medical College Thomas Jefferson University, Philadelphia, PA, USA; ^2^ Department of Urology Jefferson Einstein Medical Center, Philadelphia, PA, USA; ^3^ Department of Urology Ochsner Health System, New Orleans, LA, USA; ^4^ Department of Urology Penn State College of Medicine, Hershey, PA, USA; ^5^ New Jersey Urology, Sewell, NJ, USA

## Abstract

**Objective:**

To examine one academic institution's experiences with SpaceOAR placement, its associated complications, and periprocedural characteristics that affect outcomes for the purpose of quality improvement.

**Materials and Methods:**

We conducted a retrospective review of 233 patients who received SpaceOAR from four surgeons and one radiation oncologist between 2018 and 2021. Variables such as demographics, oncologic parameters, radiation plan, and radiographic assessment of hydrogel placement were recorded. The Charlson Comorbidity Index (CCI) was used to assess comorbidity risk. Mann–Whitney and Fisher's exact tests were performed to compare patients with and without complications.

**Results:**

Of the 233 patients who received SpaceOAR, 24 (10.3%) experienced toxicity. All complications were Clavien I or II, such as pelvic pain postplacement, pelvic fullness, bleeding, and lower urinary tract symptoms. 16 patients (6.9%) had some portion of the hydrogel injected into the rectal wall, but it was never clinically significant. The average CCI was 3.2 ± 0.95 for patients who experienced complications; the average CCI was 3.6 ± 1.6 (*p*=0.48) in the group without complications. Of the physicians with higher procedure volumes, Physician #1 had the highest rate of patient-reported complications at 11 out of 68 (16.2%) and Physician #2 had the lowest rate of complications at 4 out of 96 placements (4.2%). Multivariate analysis found that patients who had received hormone therapy previously had less odds of reporting complications after SpaceOAR placement.

**Conclusions:**

The listed attending on the procedure had a significant correlation to complications with SpaceOAR placement on univariate analysis, and hormone therapy had some benefits to the tolerance for the procedure on multivariate analysis. Overall, the hydrogel placement was well tolerated with low incidence of mild and transient procedure-related toxicity.

## 1. Introduction

Radiation therapy (RT) is a mainstay for localized or locally advanced prostate cancer. Rectal injury, exhibiting as acute and chronic proctitis, is a common concern in radiotherapeutic treatment due to the proximity of the anatomical structures, and the concern for side effects limits dose escalation.

The SpaceOAR System (Boston Scientific, Marlborough, MA USA) is an FDA-cleared implant with ultrasound-guided perineum needle insertion, and it which adds about 1.3 centimeters between the prostate and rectum decreases radiation dose to the anterior rectal wall and decreases likelihood of late rectal side effects including rectal bleeding and proctitis [1]. The substance is 90% water mixed with polyethylene glycol (PEG), which forms into a gel-like substance in vitro, and it is absorbed and excreted in the urine naturally in about 6 months. Potential complications that could arise from the placement of the hydrogel include transient symptoms, such as pain associated with injection, pain, or discomfort from the hydrogel, site inflammation, urinary urgency, constipation, rectal urgency or spasms, fainting, bleeding, infection, urinary retention, or rectal injury. More significant complications are rare but include infection, urinary retention, rectal injury, or ulcers. Severe complications are very unlikely but include the fistula, perforation, necrosis, allergic reaction (local reaction or more severe reaction, such as anaphylaxis), or embolism [1]. Studies have reported a range of complication rates with hydrogel insertion. Those finding complications as low as 0% argue report SpaceOAR as a low-risk, potential-benefit prophylactic treatment, while those finding more frequent complications as high as 10% argue that prophylaxis for RT-related rectal toxicity is no longer necessary [2–4]. One study analyzed the Manufacturer and User Facility Device Experience (MAUDE) database for all SpaceOAR-related adverse events. It argued that although complications with hydrogel injection are reportedly rare, the incidence of medical device reports increased each year, and the severity and debilitations, such as infections requiring surgical intervention, perirectal fistulae, significant bleeding, anaphylaxis requiring intensive care, and two deaths, should be considered [5]. However, Babayan et al. responded that the yearly increase in the number of medical device reports was proportional to device usage [6].

In addition, perioperative variables and the Charlson Comorbidity Index (CCI) specifically have not yet been assessed as prognostic factors. CCI is a validated method of assigning comorbidities a score from 1 to 6 based on their association with mortality and resource use. The total points are then correlated to an estimated 10-year survival and in-hospital mortality rate [7, 8]. Ghanem et al. found that CCI is a predictor of overall survival and late RT side effects including proctitis as the most prevalent gastrointestinal toxicity in prostate cancer patients [9].

We aim to elucidate the complication rate of SpaceOAR hydrogel placement and periprocedural characteristics that may factor into the outcomes so that we can improve the process at our academic hospital. In turn, we can provide patients with evidence-based information on procedural risks and benefits and potentially use perioperative factors to predict complications.

## 2. Materials and Methods

This research was not conducted under Institutional Review Board oversight as it was a quality improvement project designed not for generalizable knowledge and solely to identify and correct any processes with SpaceOAR insertion that are deviant from standard. We conducted a retrospective review of 233 patients who underwent SpaceOAR placement at our academic hospital by four surgeons (Physicians #1–4) and one radiation oncologist (Physician #5) between 2018 and 2021. SpaceOAR placements were done by four urological surgeons and one radiation oncologist. Our primary outcome was the percentage of patients experiencing complications from the procedure, and our secondary outcome was to detect some variables that could have led to the primary outcome. Variables such as demographics, medical history, oncologic parameters, and radiation plan were gathered. In addition, variables required to calculate the CCI were collected, and CCI was computed to assess preoperative comorbidity risk for each patient.

Physicians at this institution performing SpaceOAR insertion were a mix of junior and midcareer physicians. Though they had different years of experience as practicing physicians, they all started performing this procedure around the same time period. All five physicians performing the procedures had identical training with the industry representatives, including computer simulation training as well as a minimum of ten proctored procedures by an industry representative. Therefore, they all used the same instrumentation and technique disseminated by Boston Scientific [10].

Mann–Whitney tests and Fisher's exact tests were performed to compare demographics, oncological history, CCI, and other variables between those who had complications and those who did not. All statistical tests were two tailed, and *p* < 0.05 was considered statistically significant. Statistical analyses were performed using GraphPad Prism 9.0.2 (San Diego, CA).

Multiple logistic regression was performed with clinically relevant factors, which were determined based on results on univariate analyses and relevance to patient outcome before or during the SpaceOAR insertion procedure: age, BMI, physician performing the procedure, if the patient received hormone therapy prior, CCI, and if the patient was on anticoagulation therapy. Race was not in the calculations due to a lack of diversity in this patient population to include it as a predicting factor in multivariate analysis. For the categorical variables, the physician with the least complications percentage-wise, patients who did not receive hormone therapy, and patients not on anticoagulation medications were used as the baseline comparators.

## 3. Results

233 patients received SpaceOAR before radiotherapy at Thomas Jefferson University Hospital between 2018 and 2021. Physician #1 placed 68 (29.2%), Physician #2 placed 96 (41.2%), Physician #3 placed 31 (13.3%), Physician #4 placed 27 (11.6%), and Physician #5 placed 11 (4.7%).

In total, 24 of 233 (10.3%) patients experienced one or more toxicities, all of which were transient and mild, Clavien–Dindo Grades I or II. 8 (3.4%) patients experienced lower urinary tract symptoms, 1 (0.4%) experienced urinary retention requiring straight catheterization, 6 (2.6%) experienced pain, 5 (2.1%) experienced urethral bleeding, 1 (0.4%) experienced infection, and 11 (4.7%) experienced other mild complications, such as hemorrhoids, constipation, or more frequent bowel movements. 193 of the patients had posthydrogel placement imaging available, and 16 patients (8.3%) had some portion of the hydrogel injected into the rectal wall noted on MRI; however, none was clinically significant.

In comparisons between the cohort with complications and the cohort without complications, there were no significant differences in age, race, BMI, pathological staging, whether a fiducial marker was placed, type of radiation received, CCI, psychiatric conditions, or anticoagulation medications (Table 1). The only significance was found in which physician performed the hydrogel placement. Physician #4 had the highest rate of patients reporting complications after placement at 6 out of 27 patients (22.2%). Of the physicians with higher volumes of hydrogel placements, Physician #1 had the highest rate of patients reporting complications at 11 out of 68 (16.2%) and Physician #2 had the lowest patient-reported complications at 4 out of 96 placements (4.2%).

On multiple logistic regression (Table 2), there were greater odds for patients of Physician #1 (OR 4.77 [1.48–18.5], *p*=0.013) and Physician #4 (OR 4.94 [1.15–22.59], *p*=0.031) to experience complications after gel placement compared to Physician #2 who was used as the reference. However, patients that received hormone therapy had less odds than patients who did not have hormone therapy to have complications (0.38 [0.15–0.99], *p*=0.048). Age, BMI, CCI, and whether the patient was receiving anticoagulation were not significant on multivariate analyses.

## 4. Discussion

Our study showed that complications from SpaceOAR placement at our academic institution was in line with previous studies. The procedures were relatively safe with 10.7% of 233 patients experiencing transient complications similar to previous studies finding no complications with placement up to 10% in the randomized controlled Pivotal Trial [2, 3, 11].

**Figure 1:**
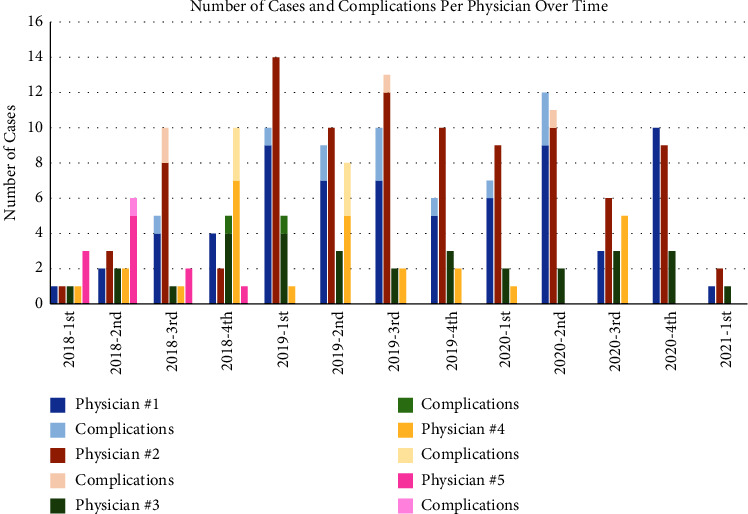
Quarterly timeline of the total number of cases and number of which one or more complications were reported per physician.

From the quality improvement standpoint, we found that there was a significant difference in the rate of patients with any complications or no complications between each physician performing the procedure. Pinkawa et al. demonstrated that there is a learning curve to hydrogel spacer placement finding significantly increased symmetry of the gel after the first 15 patients, more exclusion of the rectum from the planned target volume, and decreased dosage to the rectum [12]. They had a previous study discovering that the total procedure time decreased from 25 minutes to 14 minutes and the needle insertion time decreased from 10 minutes to 5 minutes between the first 10 and next 10 patients [13]. In our study, 4 out of 5 physicians performed greater than 15 SpaceOAR placements. The variability in procedure numbers reflected our internal practice and referral patterns. However, a learning curve was not visible based on complication rates (Figure 1). Since complications as defined in our study, such as pain and lower urinary tract symptoms, are not the most directly correlated with skill, we also looked at rectal wall injection of hydrogel as identified on MRI, and the incidences were spread throughout our study period [14].

Multivariate analysis revealed a significant difference in complications with those who received hormone therapy having lesser odds of reporting complications than those who did not receive hormone therapy. The mechanism of this finding is not clear and may not be causative, but presumably, androgen deprivation therapy (ADT) decreases the size of the prostate gland and induces tumor regression; the reduced tumor size is associated with decreased vascularity, which could improve complications such as bleeding, rectal fullness, and pain. A smaller prostate and tumor after ADT could also allow more precision during placement of the SpaceOAR gel with a more standard anatomy and more “space” in the pelvic cavity.

As with retrospective analysis of databases, there are limitations to this study. Additionally, the quality improvement goal of this project meant that the study was not systematically designed, nor did it include randomization. Our goal of comparing our institution's outcomes with the standard for this procedure can only be based on outcomes analysis from the consistency of data reported as part of care and information available in the notes. As with all procedures, benefits and risks must be weighed. The distance between the prostate and rectum has been cited one of the factors most correlated to preventing rectal toxicity [15, 16]. This would also be the measure to determine a practitioner's “successful” placement and a marker to use for monitoring and comparing between physicians. In this study we were able to assess the risks, but we did not have the measurements of the thickness of the hydrogel to quantify the benefit of the procedure. Other limitations include not having the data on the amount of time since injection until radiation, the time since injection when measurement of the SpaceOAR thickness is done, the total volume of each patient's injection by radiograph, and longer follow-up for more significant or later complications. Another limitation is the categorical nature of some of the variables collected, such as the hormone therapy where knowing the type, dosing, or length of treatment would stratify the patients and elucidate any associations, especially with the discovery that receiving ADT before SpaceOAR insertion has less odds of postprocedural complications.

With the quick advances in RT, our understanding of RT-related rectal toxicity would benefit from a randomized control trial between the different radiation modalities. Then, assessing the complications of the hydrogel placement compared to adverse events with radiation without a spacer within modalities would also guide patient care. Future studies can look at confounding factors such as the distance between the prostate and rectum pre-hydrogel placement to account for individual anatomy, comparison with institutions in which residents perform these procedures under attending supervision, their year in training, and their case logs instead of the listed attending experiences.

## 5. Conclusion

In the population of men who had SpaceOAR hydrogel placement at our academic hospital, we found that the procedure had transient and mild complications at a rate in line with previous studies. The factor that correlated most with differences between having complications or no complications was the listed attending on the procedure. However, patients who received hormone therapy had less odds of reporting complications after procedures. This will lead to more detailed investigation into the complication rates and steps for improving hydrogel placement at this academic institution.

## Figures and Tables

**Table 1:** Comparison of perioperative characteristics between patients with or without complications from SpaceOAR hydrogel placement.

	No complications (*n* = 209)	Complications (*n* = 24)	*p* value
Age	70.1 ± 6.8	71.5 ± 6.5	0.34
Race			0.67
White	129 (61.7%)	17 (70.8%)	
Black	65 (31.1%)	5 (20.8%)	
Asian	7 (3.3%)	0 (0.0%)	
Mixed race	2 (0.9%)	0 (0.0%)	
Unknown	5 (2.4%)	1 (4.2%)	
BMI	28.6 ± 5.7	26.9 ± 4.0	0.10
Physician			0.025^∗^
#1	57 (27.3%)	11 (45.8%)	
#2	92 (44.0%)	4 (16.7%)	
#3	29 (13.9%)	2 (8.3%)	
#4	21 (10.0%)	6 (25.0%)	
#5	10 (4.8%)	1 (4.2%)	
T			0.88
T1	137 (65.6%)	17 (70.8%)	
T2a	33 (15.8%)	3 (12.5%)	
T2b	18 (8.6%)	3 (12.5%)	
T2c	7 (3.3%)	1 (4.2%)	
T3a	4 (1.9%)	0 (0.0%)	
T3b	6 (2.9%)	0 (0.0%)	
N			>0.99
N0	203 (98.5%)	24 (100.0%)	
N1	3 (1.5%)	0 (0.0%)	
M			>0.99
M0	204 (100.0%)	24 (100.0%)	
M1	0 (0.0%)	0 (0.0%)	
Hormone therapy	126 (60.6%)	11 (47.8%)	0.27
Positive lesion on imaging	142 (67.9%)	18 (75.0%)	0.58
Fiducial marker	195 (93.3%)	23 (95.8%)	>0.99
Radiation type^∗^			0.96
IMRT	9 (2.8%)	1 (41.7%)	
HDR Brachy	26 (12.4%)	3 (4.2%)	
SBRT	43 (20.6%)	6 (25.0%)	
Hypofractionation	105 (50.2%)	14 (58.3%)	
VMAT	137 (65.6%)	14 (58.3%)	
Charlson Comorbidity Index	3.5 ± 1.6	3.1 ± 0.95	0.48
Psych conditions	53 (25.4%)	6 (25.0%)	>0.99
Anticoagulation medication	99 (47.4%)	14 (58.3%)	0.39

^∗^Each patient can receive more than one radiation treatment; percentages do not add up to 100.

**Table 2:** Multiple logistic regression including clinically relevant factors.

	Odds ratio (95% CI)	*p* value
Age	1.05 (0.97–1.16)	0.29
BMI	0.94 (0.87–1.03)	0.17
Physician		
#1	4.77 (1.48–18.54)	**0.013**
#3	1.82 (0.24–10.37)	0.51
#4	4.94 (1.15–22.6)	**0.031**
#5	3.77 (0.17–33.25)	0.28
Received hormone therapy	0.38 (0.15–0.99)	**0.048**
Charlson Comorbidity Index	0.74 (0.40–1.14)	0.27
Taking anticoagulation medication	0.40 (0.13–1.06)	0.08

Bolded values are *p* < 0.05.

## Data Availability

The data used to support the findings of this study have not been made available to protect patient privacy.
